# The anti-inflammatory drug Montelukast ameliorates cognitive deficits by rescuing the inflammatory levels in young AD animal models

**DOI:** 10.1038/s41598-025-91785-4

**Published:** 2025-04-13

**Authors:** Mengnan Wu, Yan-Fen Chen, Wei Yao, Siyan Zhou, Zuolei Xie, Ye Tao, Yi Zhong, Weiwei Ma

**Affiliations:** 1 School of Life Sciences, Tsinghua University, Beijing, 100084 China; 2 Beijing Joekai Biotechnology LLC, Beijing, 100094 China

**Keywords:** Alzheimer’s disease (AD), Inflammation, Proinflammatory cytokines, Cognition, Molecular biology, Neuroscience

## Abstract

Neuroinflammation precedes the clinical symptoms onset of Alzheimer’s disease (AD) by decades. However, the anti-inflammatory drugs were not always effective at all stages of the disease. Here, using the fly and mouse AD models, we evaluated the effects of anti-inflammatory drugs on inflammatory-related factors and the proinflammatory cytokines at different ages of AD animals. We also performed behavioral tests to evaluate the cognitive aspects of AD. Combined with the bioinformatics analysis, we would like to exhibit a better understanding of AD. Based on the previous studies and reanalysis of published database, we found aged AD animals might better represent the inflammatory status of symptomatic AD. Our results showed that mRNA levels of antimicrobial peptides (AMPs) were highly expressed in 10-day-old AD flies, while no significant difference was observed in 40-day-old AD. In aged APP/PS1 mice (22.5 months), inflammatory-related factors NF-κB, IBA1, and the mRNA levels of proinflammatory cytokines *Il-1β* and *Il-6* were not differentially expressed. In contrast, a significant increase was observed in 7.5-month-old APP/PS1 mice. Moreover, the anti-inflammatory drug Montelukast (MON) did not ameliorate the inflammatory and cognitive defects in 22.5-month-old aged mice but showed a rescue effect in 7.5-month-old young APP/PS1 mice. Altogether, our study demonstrates the different inflammatory status might lead to variations of anti-inflammatory drug efficacy, which helps to clarify the importance of considering the pathological stage of the disease when administering treatment.

**Supplementary Information:**

The online version contains supplementary material available at 10.1038/s41598-025-91785-4.

## Introduction

Alzheimer’s disease (AD) is the cause of more than two-thirds of all dementia cases in people aged 65 and older. The progression of AD is characterized by cognitive impairment that interferes with daily activities and observable neurodegeneration, leading to clinical manifestations of dementia. Symptoms of AD depend on the stage of the disease. AD is classified into presymptomatic, mild, and dementia-stage depending on the degree of cognitive impairment^1^. There is a long presymptomatic period between the onset of pathological changes in the brain and the development of clinical symptoms of AD^2^.

AD is a complex multifactorial disease with many contributing factors. The main neuropathological features of AD include extracellular plaques containing amyloid beta (Aβ) and intracellular neurofibrillary tangles (NFTs) formed by hyperphosphorylated tau protein, along with brain atrophy caused by the neurodegeneration of synaptic and neuronal losses^3^. In addition to these essential features, sustained activation of microglia and astrocytes is also observed in association with AD^4,5^. The activated microglia and astrocytes can produce and secrete proinflammatory cytokines, including interleukin-1α (Il-1α), interleukin-1β (Il-1β) and tumor necrosis factor-α (TNF-α), or reactive oxygen and nitrogen species, resulting in a proinflammatory response^2^. In addition, Aβ produced by neurons induces the release of the complement protein C3α by astrocytes via NF-κB signaling, which interacted with the receptors (C3αR) on microglia and neurons to aggravate Aβ aggregate loads and cognitive impairment^6^.

Growing evidence has demonstrated that neuroinflammation plays a vital role in neuropathological changes in AD^7,8^. In recent years, increasing evidence has suggested that early inflammatory changes are detectable in the presymptomatic stage, which can occur 15–20 years prior to the onset of clinical symptoms^4,9^. At this stage, the secreted proinflammatory cytokines exacerbate disease progression^10,11^. In addition, in AD patients, long-term nonsteroidal anti-inflammatory drugs (NSAIDs) ameliorate the destructive process if starting drugs intake at least 6 months before symptoms develop^12–14^. However, there are also controversial results showing no effect or even increased risk for AD^15,16^. In-depth analysis revealed that the negative effect may be due to the different stages of brain disease progression, especially in the mild or dementia stage. Thus, the pathological stage might determine the outcome of NSAID use. AD animal models are widely used for preclinical drug evaluation. Previous studies found that the young AD animal models showed the pathological defects of AD patients without neurodegeneration, while aged AD animals showed signs of neurodegeneration^17,18^, indicating the different pathological stages at different ages. Currently, no systemic investigations compared the inflammation levels and molecular characteristics at different ages in AD animal models.

In the present study, we first examined previously published database and found aged AD animal models represent better molecular characteristics of patients, and the older AD animals might represent better the inflammatory status of symptomatic AD. Then, we used an AD fly model to investigate the inflammatory levels at different ages. We found the expression of antimicrobial peptides (AMPs) were not significantly changed in aged AD flies, but significantly increased in younger AD flies, compared with the age-matched controls. The anti-inflammatory drug montelukast (MON) feeding rescued the high expression level of AMPs and cognitive defects in young AD flies. In contrast, MON did not change the expression of AMPs and showed no effects on the cognitive defects in aged AD flies. Moreover, MON even shortened the longevity of aged flies. In the mouse model, the inflammatory-related NF-κB, IBA1 and proinflammatory cytokines *Il-1β* and *Il-6* were not changed in aged AD mice, while much higher expressed in young AD, compared to the age-matched controls. Furthermore, the MON rescued the inflammatory status and cognitive defects in young mice, whereas no effects were investigated in aged AD.

## Methods

The study was carried out in compliance with the ARRIVE guidelines.

### Experimental model

#### Drosophila

The expression of secretory human wild-type Aβ42 in flies was driven by a laboratory stock of *elav-Gal4*^*c155*^ and has been previously reported^18^. Flies were raised and maintained at 60% relative humidity at 25℃ for the longevity test and at 28℃ for the pavlovian olfactory associative immediate memory test. All the flies were kept on standard cornmeal food supplemented with antibiotics under a 12-h light/dark cycle. All stocks used for Pavlovian olfactory conditioning were equilibrated by five generations of outcrossing to the isogenic line w^1118^ (isoCJ1).

#### Mice

We used the double transgenic mice that express a mutant chimeric mouse/human APPswe and a mutant human presenilin 1 (Delta E9), both driven by the prion protein promotor, were purchased from the Jackson Laboratory (strain B6C3-Tg (APPswe.PSEN1dE9) 85Dbo/Mmjax)^19^. Male and female littermates were gender-balanced and randomly assigned to vehicle control or drug treatment groups. All mice were housed at a constant 23℃ under a 12-h light/dark cycle, with food and water available *ad libitum*. Mice were single housed. All animal procedures were conducted under the guidance and approval of the Institutional Animal Welfare and Ethics Committee of Tsinghua University, and they conformed to the National Institutes of Health guidelines on the ethical use of animals.

### AD cohort and concordant analysis

For comparisons of gene expression changes in human AD and mouse models of AD, we utilized differentially expressed genes (DEGs) data of the AD Cross Species Study cohort (10.7303/syn16779040). In the cohort, they examined 30 AD-associated, gene co-expression modules, which were generated separately by the Accelerating Medicines Partnership^®^ Program for Alzheimer’s Disease (AMP-AD) consortium. The 30 AD-associated human brain co-expression modules were overlapped with 234 curated DEG sets from mouse brain RNA-sequencing experiments, reprocessed from 99 studies in total. The overall pipeline for these analyses included (1) RNA-seq reprocessing, (2) differential expression analysis, and (3) mouse-human overlap analysis. DEGs were identified using a threshold of false-discovery rate (padj) of 1% and minimum of 1.2-fold change. All DEG analyses were completed with R v3.4.2, and Python v.2.7.12. We downloaded the processed DEGs table of the APP/PS1, and TgCRND8 mouse models at different ages from Synapse (only these two AD models contain mouse data of the same genotype at different ages) and calculated the proportion of concordant/discordant changes by dividing the number of concordant/ discordant changes to the sum of concordant and discordant changes.

### Quantitative reverse-transcription PCR

Total RNA was isolated from 30 fly heads using Direct-zol RNA Miniprep Plus Kits (ZYMO Research, #R2072) or from 10 mg of mice cortical tissue with Direct-zol RNA Miniprep Kits (ZYMO Research, #R2050) according to the manufacturer’s instructions. The first strand cDNA was then transcribed with the SuperScript III First-Strand Synthesis SuperMix (Invitrogen, #18080400). Quantitative PCR was performed on a StepOnePlus Real-Time PCR System (Applied Biosystems) using SYBR Master Mix (Invitrogen) to measure amplification. The levels of all genes were normalized to those of the housekeeping gene Rp49 (drosophila) or 18s rRNA (mice) in RT-PCR and expressed as fold changes relative to the relevant controls. The primers used in this study are listed in Table S1.

### Drug administration

Files

MON (Selleck, #S4211) and sasapyrine (Selleck, #S4188) were dissolved in dimethyl sulfoxide (DMSO) to prepare 100X stock solutions, which were stored at -20℃. All the solutions were further diluted in 4% sucrose to the final concentrations of 100 µM MON and sasapyrine. Flies were starved for 2 h in empty vials and fed drugs or vehicle (50 µl). Then, they were transferred to normal food after each treatment. Drug feeding was carried out once each day during the treatment period.

Mice

Mice were treated with 2 mg/kg (body weight) MON (in CMC-Na, 4 weeks, by gavage)^20^, or CMC-Na (vehicle) once a day followed by the Morris water maze (MWM) tests. Some old APP/PS1 mice died during drug treatment or the MWM test, so the number of mice after the MWM test might have been less than the number at the start of drug treatment. All animal work complied with ethical regulations for animal testing and research and was done in accordance with Institutional Animal Care and Use Committee approved by Tsinghua University. The animal protocol number is 18-ZY1.

### Behavioral tests

#### Pavlovian olfactory associative immediate memory

The training and testing procedures were the same as previously described^19,21^. During one training session, a group of 100 flies was sequentially exposed for 60 s to two odors, 3-octanol (OCT; Fluka) or 4-methylcyclohexanol (MCH; Fluka), with 45 s of fresh air between presentations. Flies were subjected to foot-shock (1.5-s duration with 3.5-s interstimulus interval, 60 V) during exposure to the first odor (CS+) but not the second odor (CS-). To measure “immediate memory” (also referred to as “learning”), the flies were transferred immediately after training to the choice point of a T-maze and forced to choose between the two odors for 2 min. Then the flies were trapped in their respective T-maze arms, anesthetized with carbon dioxide, and counted. A performance index was calculated from the distribution of this group of flies in the T-maze. A reciprocal group of flies was trained and tested by using OCT as the CS+ or MCH as the CS+. The so-called half-performance index, performance index (OCT) and performance index (MCH), were finally averaged and multiplied by 100, yielding an *n* = 1. A performance index of 0 indicated a distribution of 50:50 (no learning), whereas a performance index of 100 indicated “perfect learning”: 100% of the flies avoided the CS+ previously paired with foot shock. The control groups were age-matched to the experimental groups in each test.

#### Morris water maze

The MWM test was adopted and modified from previous reports^19,22,23^. Briefly, a round tank (120 cm in diameter) was filled with water (19–20℃) that was made opaque with milk. A transparent platform (15 cm in diameter) was placed in the center of one of the four virtually divided quadrants and 2 cm below the water surface. Distal cues were provided in all experiments as spatial references. Mice were put into the tank in another quadrant and allowed to swim until they found the platform and remained on it for at least 5 s. During the first day of the experiment (day 1), if a mouse did not find the platform within 60 s, the mouse was gently guided to the platform and remained there for 5-s stay. From days 2–5, mice did not find the platform within 60 s, they were given a latency of 60 s. Mice were trained four trials per day. The latency to find the platform was recorded for each trial and the four daily trials were averaged for statistical analysis. Before (day 0) and after training, day 6 (probe trail), the platform was removed, and mice were allowed to swim for 60 s. The time spent in the target quadrant was recorded. A video tracking system (Jiliang Software Technology, China) was used to automatically record the swimming route and analyze the data.

#### Fly longevity experiment

On the first day after eclosion, 50 male flies were selected and transferred to fresh food vials, and then placed in a 25 ℃ incubator until drug feeding started. The medium was replaced every two days and death events were recorded at the same time until at least all the flies in one group died. The lifespan experiments were independently performed at least three times and each group contained at least 4 vials each time.

### Western blotting

Cortical brain tissue was lysed using cell lysis buffer (Beyotime, #P0013J) supplemented with protease and phosphatase inhibitor cocktails (Selleck), after which the protein concentration was quantified with a BCA protein quantification kit (Beyotime). Proteins were separated by SDS-PAGE and transferred onto a nitrocellulose membrane (Millipore). Membranes were probed with the specific primary antibodies, followed by peroxidase-conjugated secondary antibodies. The bands were visualized by Sage Brightness Plus (Sinsage, #Q02003). The imaging data were analyzed with ImageJ 6.0 (National Institutes of Health). The primary antibodies used were as follows: Rabbit anti-NFκB (Cell Signaling Techonology, #8242), Rabbit anti-IBA1 (Cell signaling Technology, #17198), Mouse anti-actin (Biodee, #DE0620). The secondary antibodies were as follows: goat anti-rabbit (Cell Signaling Technology, #7074), and donkey anti-mouse (Cell signaling, #7076), HRP-linked antibodies.

### Quantification and statistical analysis

All datasets were analyzed using GraphPad Prism 7 or SPSS 20.0. Comparisons between three or more independent groups were performed using one-way ANOVA with Tukey HSD post-hoc multiple comparison test. Two-tailed unpaired student’s *t-*tests were used to compare two groups. The results of the longevity experiments were analyzed using log-rank (Mantel-Cox) test. Escape latency during training of MWM was analyzed using repeated measures of ANOVA test. Data are presented as the mean ± SEM. Statistical significance was evaluated at *P* < 0.05.

## Results

### Similarities in gene expression of AD animal models at different ages with symptomatic patients

**Fig. 1:**
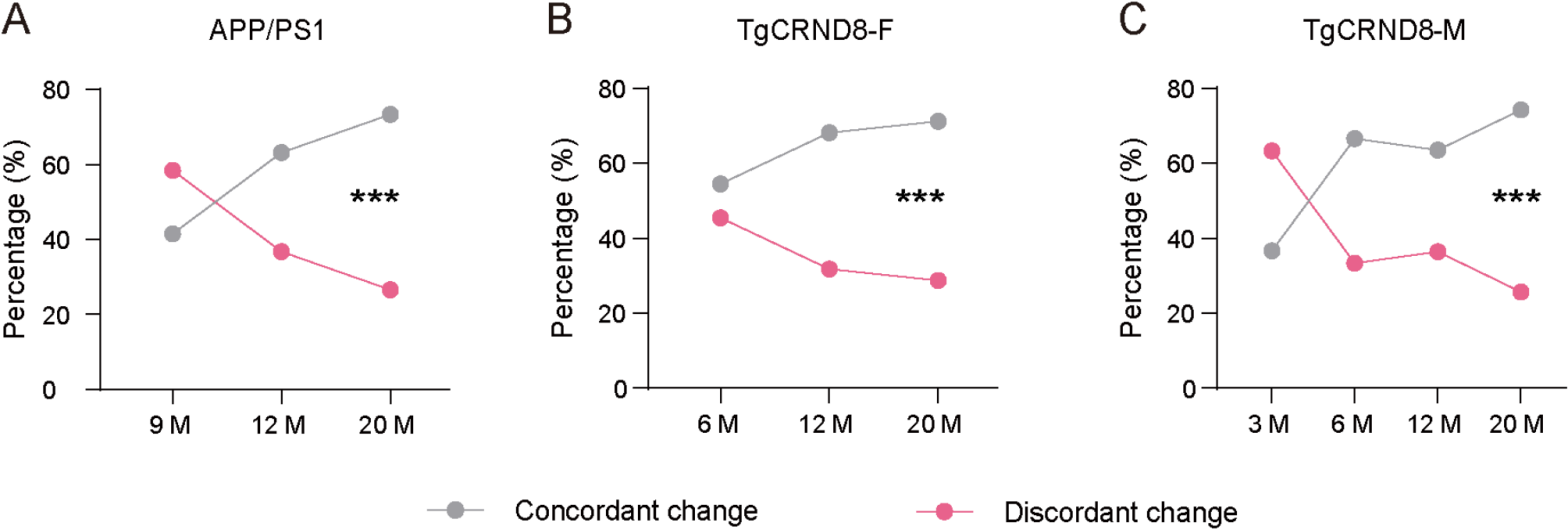
The similarity of molecular changes between symptomatic AD patients and AD mouse models at different ages. (**A-C**) The concordant and discordant changes between the APP/PS1 (**A**), female (**B**) and male (**C**) TgCRND8 AD mouse models and symptomatic AD patients (Chi-square test, APP/PS1: χ² = 97.13, *P* < 0.001; TgCRND8-F: χ² = 424.8, *P* < 0.001; TgCRND8-M: χ² = 94.75, *P* < 0.001). *** *P* < 0.001.

The animal model of the AD is an important tool for mechanistic studies and therapeutic evaluation. Although younger transgenic AD mouse models widely used in the study are able to simulate the pathological and phenotypic defects of AD in many aspects, including Aβ protein deposition, and learning and memory deficits, they do not exhibit neuronal loss or brain atrophy like AD patients^17^; however, older transgenic AD models, such as the APP/PS1 model over 17–22 months of age and the APP/PS1/tau model over 12 months of age, do show signs of neurodegeneration^17^. This indicates that older AD models are better at simulating the pathological characteristics of patients with neurodegeneration. To explore the similarities of the molecular characteristics of AD mouse models at different ages with those of AD patients, we conducted a search on the “AD Knowledge Portal” (https://adknowledgeportal.synapse.org/), which shares data and resources generated by multiple collaborative research projects, including large amounts of genomic variations and transcriptional data from studies of aging, dementia, and AD^24^. After searching, we found a cross-species study (The AD Cross Species Study (AD_CrossSpecies)) under the program of the AMP-AD. In this study, they obtained transcriptomic data from the brain tissue of symptomatic AD patients and healthy individuals and identified 30 differentially expressed gene modules. Then, the transcriptional data of AD mouse models and other neurodegenerative mouse models were collected. The APP/PS1 and TgCRND8 mice are two representative AD models overexpress APP and PS1 or only APP proteins, respectively. We found that the proportion of concordant changes increased with age, whereas the proportion of discordant changes decreased with age in the APP/PS1 and TgCRND8 mouse models (Fig. 1A-C; Table 1). We further analyzed the differences in molecular characteristics of AD mice across different age groups. The null hypothesis (H_0_) states that molecular characteristics are independent of mice’s ages. Our results revealed significant differences in molecular characteristics among different ages in APP/PS1 (Fig. 1A), and in both female (Fig. 1B) and male (Fig. 1C) TgCRND8 AD mouse models. These results suggested that the molecular characteristics of aged transgenic mice are more similar to those of symptomatic AD.

**Table 1:** The concordant and discordant changes in the cross-species study.

Mouse strains	The concordant changes	Concordant change (%)	The discordant changes	Discordant change (%)
APP/PS1_9 month	183	41.50	258	58.50
APP/PS1_12 month	74	63.25	43	36.75
APP/PS1_20 month	348	73.42	126	26.58
TgCRND8_F_6 month	5086	54.47	4251	45.53
TgCRND8_F_12 month	1776	68.15	830	31.85
TgCRND8_F_20 month	3234	71.26	1304	28.74
TgCRND8_M_3 month	11	36.67	19	63.33
TgCRND8_M_6 month	505	66.62	253	33.38
TgCRND8_M_12 month	2516	63.58	1441	36.42
TgCRND8_M_20 month	1923	74.25	667	25.75

### The inflammatory status in symptomatic AD patients

Neuroinflammation, a critical cellular system in AD, has received extensive attention in clinical research and development^1^. However, several trials have shown the lack of efficacy of NSAIDs for treating AD after clinical symptoms^25–27^. Thus, we reanalyzed the RNA-seq data of 402 cytokine genes from the “Religious Orders Study/Memory and Aging Project (ROSMAP). We found that the levels of most cytokines in the dorsal prefrontal cortex (PFC) were not significantly different between the symptomatic AD patients and age-matched healthy controls. Specifically, the expression levels of the proinflammatory cytokines interleukin 1β (IL-1β) and interleukin 6 (IL-6) did not show any significant changes among the normal control healthy (NC), mild cognitive impairment (MCI) and dementia stage of AD patients (Table 2). Thus, the RNA level of proinflammatory cytokines of IL-1-1β and IL-6 are not differently activated between the symptomatic AD patients and controls. However, significant upregulation of IL-6, IL-1β, TNF-α, and NF-κB levels has been observed at 6-9-month-old APP/PS1 and 5X FAD mice^28–30^. Studies on older AD mouse models are not yet sufficient, but limited research has found that in older AD mouse models (15–18 months old), the levels of IL-6, IL-1β, and NF-κB are not significantly different from those in age-matched wild-type mice^31^.Taken together, these results revealed that older AD animals might represent better the inflammatory status of the symptomatic AD.

**Table 2:** The mRNA expression level of inflammatory cytokines in the prefrontal cortex in patients with AD during the MCI and dementia stage.

Cytokine	NC	MCI	AD	F_2, 588_ value	*P* value
IL-1β	0.84 ± 0.10	0.77 ± 0.08	0.84 ± 0.20	0.07	0.93
IL-6	1.17 ± 0.23	1.05 ± 0.21	1.07 ± 0.12	0.12	0.88

Data are shown as Mean ± SEM.

### Anti-inflammatory effects on young, but not aged AD flies

**Fig. 2:**
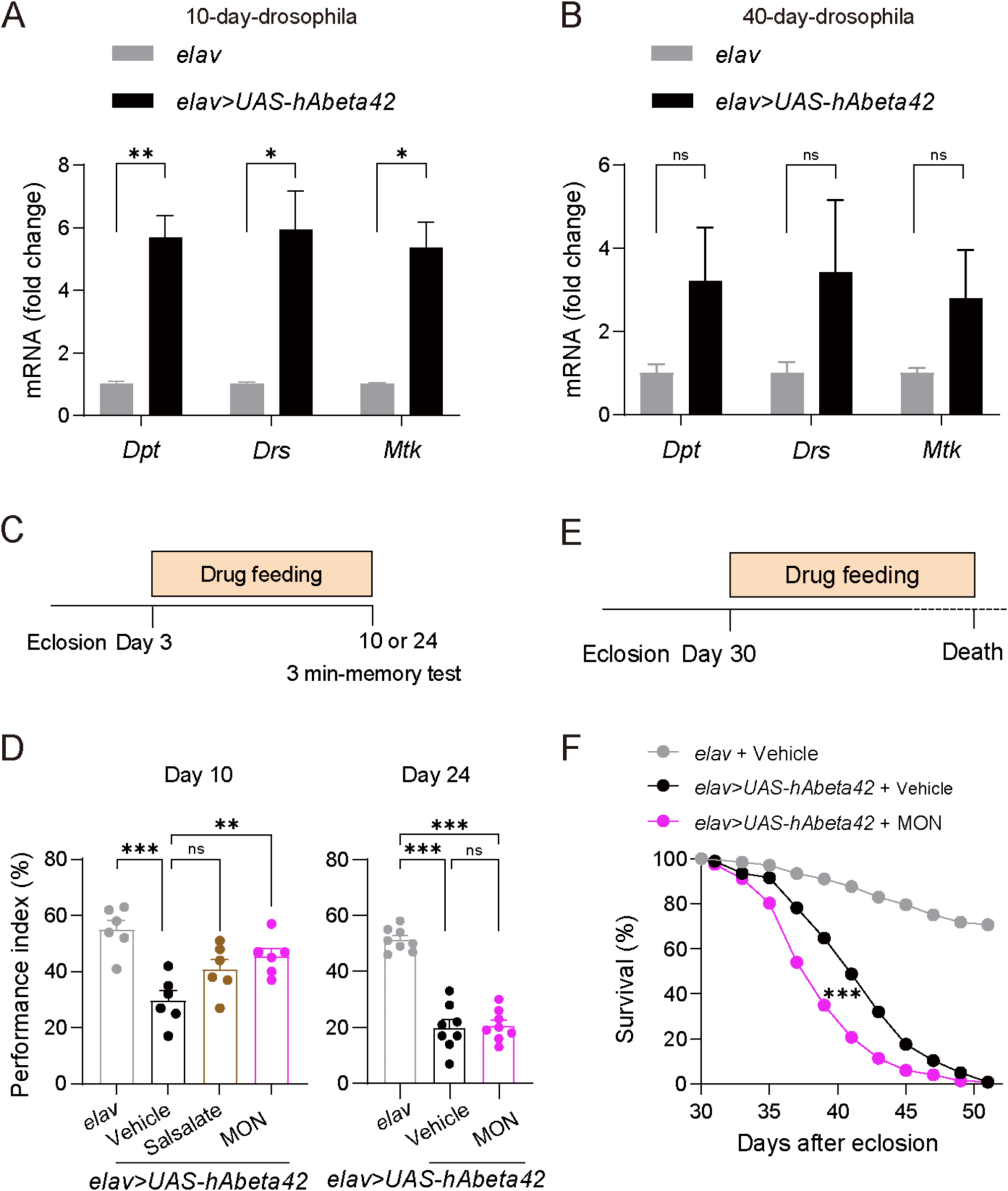
The treatment effect of anti-inflammatory drugs on different ages of AD flies. (**A**) Aβ induced AMPs overexpression in 10-day-old flies. (**B**) Aβ did not induce significant AMPs overexpression in 40-day-old flies. (**A** and **B**, unpaired student’s *t*-test). (**C-D**) Schematic (**C**) and quantification (**D**) of the performance index for 10-day-old (left) and 24-day-old (right) flies treated with the anti-inflammation drugs sasapyrine or MON. Day 10: *n* = 6; Day 24: *n* = 8 (One-way ANOVA: Day 10, *F*_3,20_ = 10.06, *P* = 0.0003; Day 24, *F*_2,21_ = 67.96, *P* < 0.001). (**E-F**) Schematic (**E**) and quantification (**F**) of the survival probability for 24-day-old flies treated with MON. *elav +* vehicle, *n* = 347; *elav* > *UAS-hAbeta 42* + vehicle, *n* = 348; *elav* > *UAS-hAbeta 42* + sasapyrine, *n* = 354 flies (log-rank Mantel-Cox test: χ² = 631.9, *P* < 0.001). Data are expressed as mean + SEM. ** *P* < 0.01; *** *P* < 0.001; ns, not significant.

Next, we investigated the inflammatory signaling in young and aged flies. We used an h-Ab42wt-expressing fly model that recapitulates many clinical features of AD, including memory loss, movement deficiency, neurodegeneration and a shortened life span^18^. In *Drosophila*, antimicrobial peptides (AMPs) are produced downstream of Toll and Imd NF-κB pathways upon infection^32,33^. We found that the mRNA expression levels of AMPs, including Diptericine (*Dpt*), Drosomycin (*Drs*) and Metchnikowin (*Mtk*), were significantly increased in 10-day-old AD flies (Fig. 2A). Unlike that of AMPs in young flies, the mRNA expression level was not significantly influenced in 40-day-old AD flies (Fig. 2B). We next examined whether anti-inflammatory drugs might also selectively improve cognition in young AD flies. For this purpose, we fed flies with the anti-inflammatory drug of MON or sasapyrine (Fig. 2C; Figure S1A) before starting the behavioral test. We found that 10-day-old flies fed MON exhibited an increase in the performance index, whereas 24-day-old AD flies fed MON did not exhibit an increase in the performance index, compared to the AD control flies (Fig. 2D). Moreover, we found that AD flies fed with sasapyrine did not exhibit any improvement at either 10 or 24 days of age (Fig. 2D; S1B). Furthermore, we investigated longevity after feeding with the MON or sasapyrine starting at 30 days after eclosion (Fig. 2E; Figure S1C). We found no positive effects of MON treatment, but MON treatment even shortened the life span of AD flies (Fig. 2F). Additionally, we observed no beneficial effect of sasapyrine treatment on the lifespan of AD flies (Figure S1D). Thus, the beneficial effects induced by anti-inflammatory drugs were only effective in young flies.

### Anti-inflammatory effects on young, but not aged AD mice

**Fig. 3:**
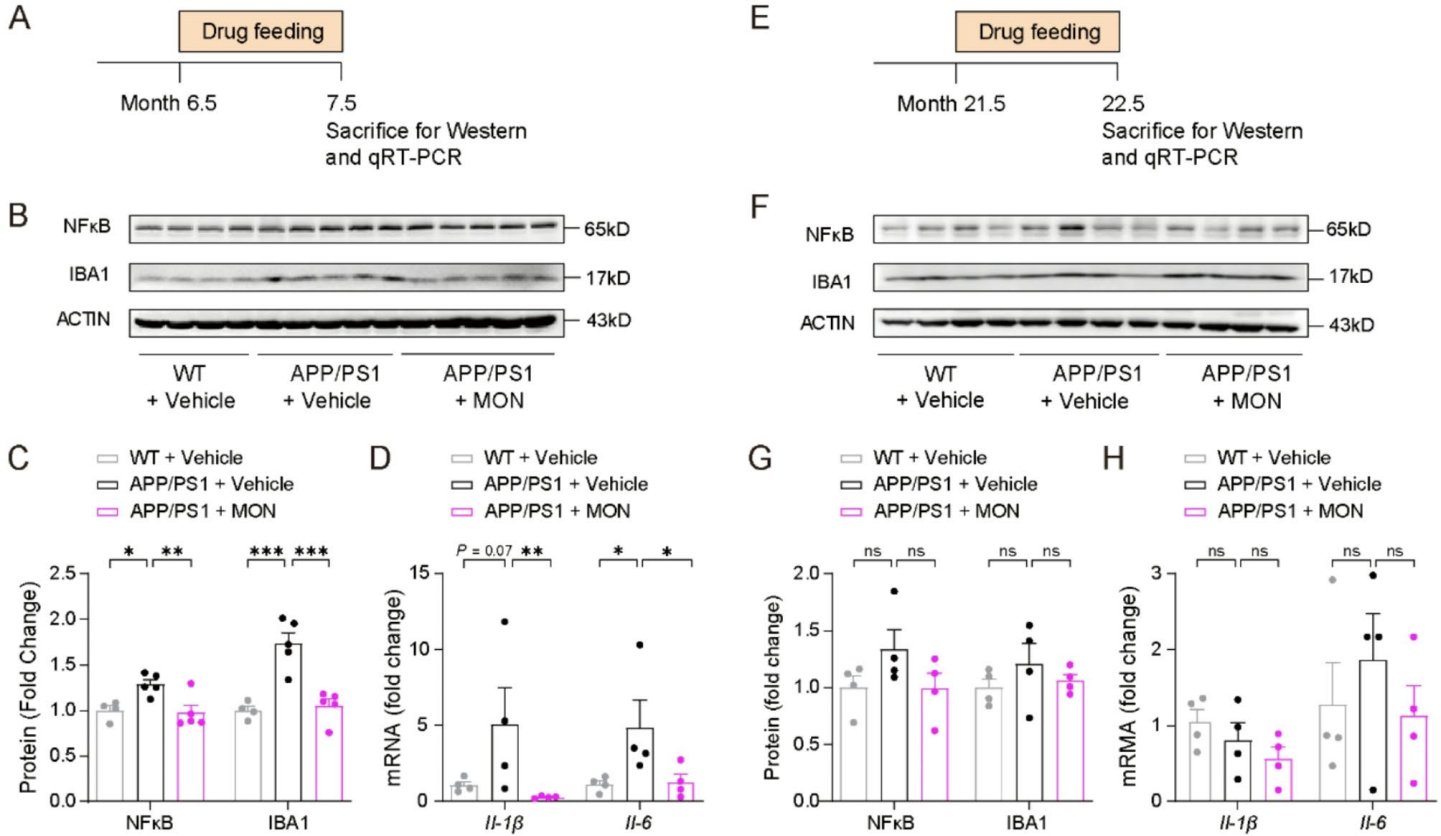
Validation the effect of MON on inflammatory responses in AD mice. (**A**) Timeline of MON treated in the young AD mice. (**B-C**) Immunoblots (**B**) and analysis (**C**) of NFκB and IBA1 in the cortex of 7.5-month-old AD mice. WT + Vehicle, *n* = 4; APP/PS1 + Vehicle, *n* = 5; APP/PS1 + MON, *n* = 5, one-way ANOVA, NFκB: *F*_2,11_ = 7.27, *P* = 0.01; IBA1: *F*_2,11_ = 20.38, *P* = 0.0002. (**D**) The mRNA expression of *Il-1β* and Il-6 in the cortex of 7.5-month-old AD mice. WT + Vehicle, *n* = 4; APP/PS1 + Vehicle, *n* = 4; APP/PS1 + MON, *n* = 4, one-way ANOVA, *Il-1β*: *F*_2,9_ = 3.33, *P* = 0.08; *Il-6*: *F*_2,9_ = 3.56, *P* = 0.07. (**E**) Timeline of MON treated in aged AD mice. (**F-G**) Immunoblots (**F**) and analysis (**G**) of NFκB and IBA1 in the cortex of 22.5-month-old AD mice. *n* = 4 per group, one-way ANOVA, NFκB: *F*_2,9_ = 1.95, *P* = 0.20; IBA1: *F*_2,9_ = 0.84, *P* = 0.46. (**H**) The mRNA expression of *Il-1β*, *Il-6* in the cortex of 22.5-month-old AD mice. *n* = 4 per group, one-way ANOVA, *Il-1β*: *F*_2,9_ = 1.69, *P* = 0.24; *Il-6*: *F*_2,9_ = 0.55, *P* = 0.59. **B** and **F** cropped images are displayed, uncropped immunoblots are displayed in Figure S4. Data are expressed as mean + SEM. * *P* < 0.05; ** *P* < 0.01; *** *P* < 0.001; ns, not significant, compared to the APP/PS1 + Vehicle group.

To further validate these findings, we conducted a study in an AD mouse model. We used APP/PS1 double transgenic mice, which express two familial AD-linked mutants, human amyloid β precursor protein (APPswe) and human presenilin 1 (Delta E9)^34^. The double transgenic mice exhibited extensive Aβ-overexpressing plaques, reduced behavioral impairment, neuroinflammation, and neuronal degeneration^17,35^. Previous studies have indicated that MON plays a beneficial impact in cognitive deficits and reduces neuroinflammation in a drug-induced AD mouse model^20,36,37^. We treated mice with MON for 4 weeks before performing western blotting to quantify NF-κB and IBA1, which are key transcription factors that modulate inflammatory responses and markers of microglia, respectively (Fig. 3A and E). We found that the 7.5-month-old APP/PS1 mice had significantly greater expression of NF-κB and IBA1 in the cortex than the WT controls, and MON administration attenuated this increase (Fig. 3B and C). In contrast, the two proteins were not significantly altered in 22.5-month-old APP/PS1 mice, and MON did not change the protein level in the cortex (Fig. 3F and G). Moreover, the mRNA levels of the inflammatory cytokines *Il-1β* and *Il-6* were determined by qRT-PCR after 4 weeks of MON administration (Fig. 3A and E). Compared to the age-matched WT control mice, the 7.5-month-old APP/PS1 mice showed an increased tendency (*p* = 0.07) of *Il-1β* and a significant increase in *Il-6*, which were rescued by MON treatment (Fig. 3D). Furthermore, *Il-1β* and *Il-6* were not affected in the 22.5-month-old mice, while the MON did not significantly change their mRNA levels (Fig. 3H). These results suggested that the anti-inflammatory drug MON is selectively capable of rescuing the inflammatory defects in young AD mice, but not in aged AD mice.

### MON did not ameliorate Spatial learning and memory in 22.5-month-old AD mice

**Fig. 4:**
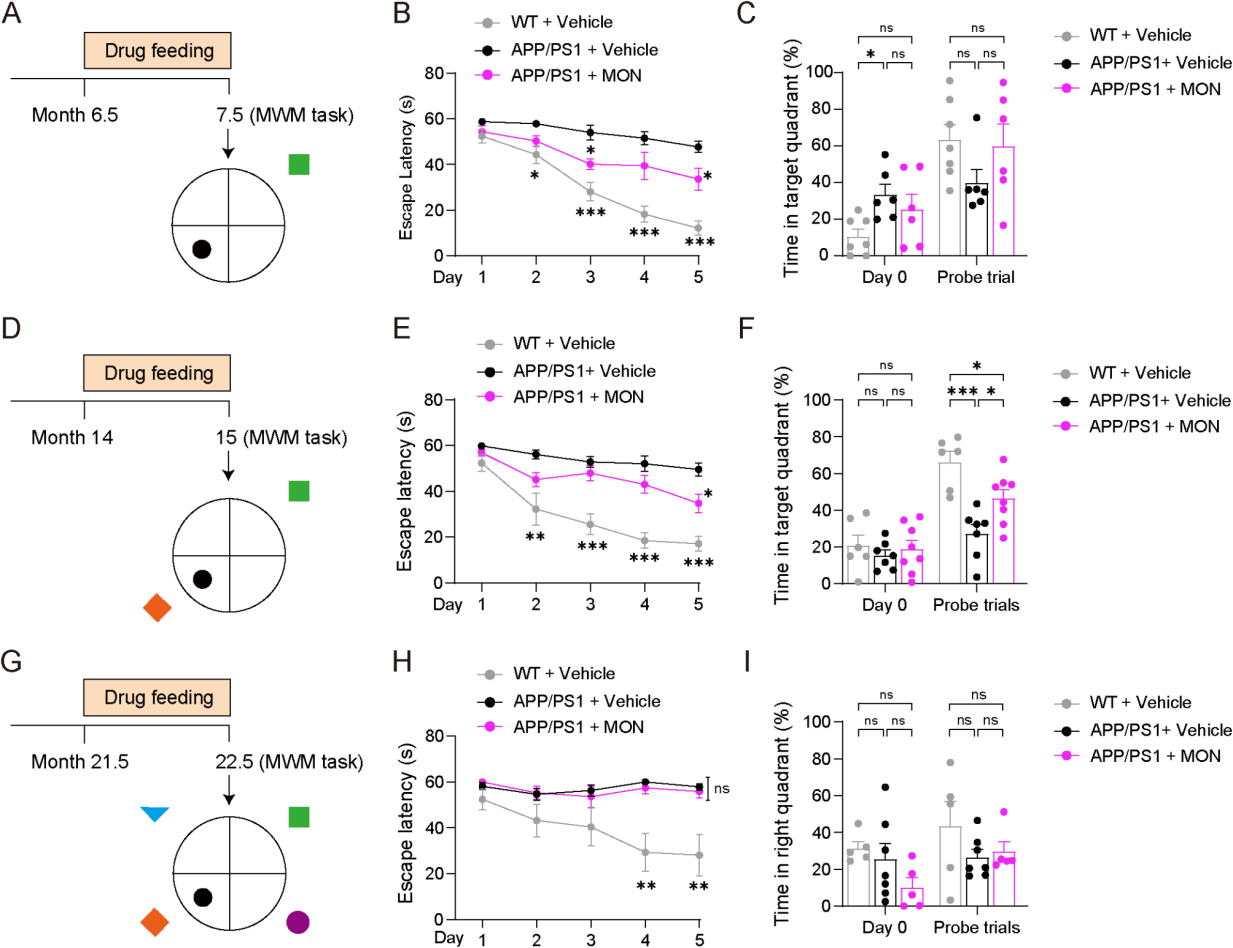
MON did not ameliorate the spatial learning and memory in 22.5-month-old AD mice. (**A**) Schematic illustration of the MWM task of 7.5-month-old AD mice. A cue was placed on one quadrant. (**B**) The escape latency during training trial. Day effect: *F*_4,64_ =27.44, *P* < 0.001; group effect: *F*_2,16_ = 47.25, *P* < 0.001; Day x group: *F*_8,64_ = 4.08, *P* < 0.001. (**C**) The amount of time spent in the target quadrant when the platform was absent for 7.5-month-old AD mice treated with MON. Day 0: *F*_2,16_ = 3.97, *P* = 0.04. Probe trials: *F*_2,16_ = 1.79, *P* = 0.20. (**D**) Schematic illustration of the MWM task of 15-month-old AD mice. Two cues were placed in opposite quadrants. (**E**) The escape latency during training trial. Day effect: *F*_4,72_ = 26.59, *P* < 0.001; group effect: *F*_2,18_ = 27.33, *P* < 0.001; Day x group: *F*_8,72_ = 3.74, *P* = 0.001. (**F**) The amount of time spent in the target quadrant when the platform was absent for 15-month-old AD mice treated with MON. Day 0: *F*_2,18_ = 0.34, *P* = 0.72; Probe trials: *F*_2,18_ = 13.29, *P* = 0.0003. (**G**) Schematic illustration of the MWM task of 22.5-month-old AD mice. Four cues were placed on the quadrants. (**H**) The escape latency during training trial. Day effect: *F*_4,56_ = 3.04, *P* = 0.024; group effect: *F*_2,14 =_ 12.31, *P* < 0.001; Day x group: *F*_8,56_ = 2.74, *P* = 0.013. (**I**) the time spent in the target quadrant when the platform was absent of 22.5-month-old AD mice treated with MON. Day 0: *F*_2,14_ = 2.203, *P* = 0.15. Probe trials: *F*_2,14_ = 1.22, *P* = 0.32. Month 7.5: WT + Vehicle, *n* = 7; APP/PS1 + Vehicle, *n* = 6; APP/PS1 + MON, *n* = 6; Month 15: WT + vehicle, *n* = 6; APP/PS1 + Vehicle, *n* = 7; APP/PS1 + MON, *n* = 8. Month 22.5: WT + vehicle, *n* = 5; APP/PS1 + Vehicle, *n* = 7; APP/PS1 + MON, *n* = 5. **B**, **E** and **H**: Repeated measures of ANOVA test with Tukey HSD post-hoc multiple comparisons. **C**, **F** and **I**: One-way ANOVA. Data are expressed as mean + SEM. * *P* < 0.05; ** *P* < 0.01; *** *P* < 0.001; ns, not significant, compared to the APP/PS1 + Vehicle group.

Finally, we determined whether MON treatment exerts different effects on cognition in AD mice of different ages. The elevated accumulation of Aβ, hyperphosphorylated tau and neuronal loss were found in hippocampus and cortices^38,39^. Pathological patterns of hippocampus and cortices have been shown to track disease progression^40,41^. To address the cognition effect, the MWM paradigm was used to evaluate the hippocampus-dependent spatial learning and memory. We settled the number of distinct cues to change the difficulty of the MWM task. One cue was settled to investigate spatial learning and memory for 7.5-month-old mice (Fig. 4A). The results showed that all groups of mice learned this task after 5 days of training, as the escape latency between day 1 and day 5 was significantly different in 7.5-month-old groups (Figure S2A). Specifically, the 7.5-month-old APP/PS1 mice spent significantly more time seeking the hidden platform from day 2 to 5 during the training phase, compared to WT mice (Fig. 4B), despite already showing a preference for the target quadrant before training (Fig. 4C). Overall, our findings indicated that continuous training did not improve the learning deficiency of APP/PS1 mice. In contrast, APP/PS1 mice treated with MON spent less time seeking the hidden platform on day 3 and day 5, compared to those treated with the vehicle (Fig. 4B). However, APP/PS1 mice treated with MON did not rescue the performance in the final probe trial, although the time spent in the target quadrant rebounded to the direction of the WT mice (Fig. 4C). All these results indicated that the 7.5-month-old APP/PS1 mice had severe deficits in spatial learning and memory. Such learning deficiency was significantly rescued after the administration of MON treatment on day 3 and 5 (Fig. 4B).

Furthermore, we used two cues to indicate spatial information for 15-month-old mice to perform the MWM test due to learning and memory deficits with aging (Fig. 4D). The escape latency on day 1 and day 5 showed significant difference in all three groups, indicating mice were able to learn the task after 5 days of training (Figure S2B). Moreover, the results indicated that APP/PS1 mice showed learning deficiencies from day 2 to 5 during the training phase, compared to the WT mice (Fig. 4E). MON treatment ameliorated the spatial learning ability; that is, it significantly shortened the time spent seeking the hidden platform on the final day of training (Fig. 4E). Moreover, the MON rescued spatial memory by significantly increasing the time spent in the target quadrant in 15-month-old APP/PS1 mice (Fig. 4F). Thus, the anti-inflammatory drug of MON appears to have a positive effect on learning and memory in younger AD mice (7.5 and 15 months).

For 22.5-month-old mice, we first tested the MWM under two cues (Figure S3A). We found no difference between the APP/PS1 mice and WT mice in terms of escape latency (Figure S3B), and time spent in the target quadrant during the probe trial (Figure S3C), suggesting that the MWM paradigm presented a cognitive challenge that exceeded the capabilities of aged mice. Therefore, we adapted the MWM test by introducing four distinct cues as spatial references to assess spatial learning and memory capabilities (Fig. 4G). As indicated, WT mice successfully learned the task, showing a significant difference in escape latency between day 1 and day 5. In contrast, APP/PS1 mice did not exhibit a significant change in escape latency, even with MON treatment (Figure S2C). The APP/PS1 mice spent much more time seeking the hidden platform during the learning phase on day 4 and 5, and MON treatment did not affect their learning ability (Fig. 4H). Moreover, MON was unable to improve the memory loss phenotype of APP/PS1 mice in the probe trial (Fig. 4I). Taken together, these results suggested that the anti-inflammatory drug is selectively capable of rescuing cognitive deficits in young AD mice, but not in aged AD. This distinction might be due to the variations of their inflammatory status. This lack of efficacy of MON in aged AD mice raised questions about the efficacy of anti-inflammatory drugs in symptomatic patients.

## Discussion

In the present study, we demonstrated the different efficacy of anti-inflammatory drug in young and aged AD animal models. We first showed that aged AD animal models exhibit better molecular characteristics of patients, and the expression levels of inflammatory factors and proinflammatory cytokines were much greater in young animals but were not significantly changed in aged AD animals. We then applied the anti-inflammatory drug to AD animals and found a beneficial effect in young, but not in aged AD animals. These findings are consistent with the long-term NSAIDs research indicating beneficial effects for early intervention before symptoms, but negative or no effects for mild or dementia AD patients^14,15,42^.

Given that animal models of AD are crucial tools for mechanistic research and therapeutic evaluation. The persistently high failure rate of drug development has led to skepticism regarding these animal models. Despite the widespread use of young transgenic AD mouse models (7–8 months old) in animal studies, which can mimic the pathological defects of AD in terms of Aβ deposition and NFT tau pathology, they did not exhibit neurodegeneration. In contrast, older transgenic AD models, such as the APP/PS1 model over 22 months of age, and the APP/PS1/tau model over 12 months of age, show signs of neurodegeneration^17^. These findings indicate that older AD models are better at simulating the pathological characteristics of patients with respect to neurodegeneration. In several AD mouse models, the significant upregulation of Il-1β, Il-6, TNF-α and NFκB was investigated in young mice (6–9 months old)^28–30^. Although, there are insufficient inflammation-related studies on aged AD mouse models, the limited studies found no significant differences in Il-1β, Il-6, NF-κB levels in aged AD mouse models (15–18 months old) compared to the wild-type mice^31^. Currently, no systemic study has compared the molecular characteristics of AD animal models with those of AD patients. We conducted the investigation and reanalysis of AD animal models and symptomatic patients. Our findings indicated that the aged AD mice represent better of the symptomatic patients. Next, we performed an in-depth investigation of the widespread attention given to neuroinflammatory responses in AD-related research. The expression levels of Il-1β, Il-6 and TNF-α are quite controversial; some studies have shown increases in the CSF^43^, while others have shown no significant up-regulation^44^. YKL-40, another well-characterized pro-inflammatory cytokine and a biomarker for early glial activation and AD diagnosis^45,46^. A meta-analysis indicated that YKL-40 expressed higher in the cerebrospinal fluid (CSF) of symptomatic AD patients compared to age-matched healthy controls. In contrast, the expression of pro-inflammatory cytokines IL-1β, IL-6, and tumor necrosis factor α (TNF-α) was not significantly upregulated in symptomatic patients^44^. Since the levels of inflammatory cytokines in CSF might not be able to reflect changes at the brain tissue level, we utilized data analyzed from ROSMAP data and found no upregulation of Il-1β, Il-6 and TNF-α at the mRNA level in the prefrontal cortex in symptomatic AD. Thus, young AD mouse models may not fully represent the inflammatory state of symptomatic AD. Conducting more comprehensive studies on both young and aged AD mouse models could lead to a deeper understanding of the inflammatory mechanisms involved in symptomatic AD.

Growing evidence indicates that the inflammation plays a vital role in the neuropathological changes in AD^47^. The proinflammatory cytokines activate the phosphatidylinositol-specific phosphatase C (PLC) and induce the production of inositol triphosphate (IP3) and diacylglycerol (DAG), which then activate protein kinase C (PKC) to produce leukotrienes^48^. The inflammatory actions of leukotrienes are mediated via cell-surface receptors, which are mainly classified into two subtypes, termed the cysteinyl leukotriene receptor (CysLT1R) and CysLT2 receptors^49^. In our study, we detected increased expression levels of AMPs and inflammation-related factors and proinflammatory cytokines in young AD animals. To investigate the therapeutic effects of anti-inflammatory drug on AD animal models of different ages, we employed MON, a highly selective antagonist of CysLT1R, which primarily exerts its pharmacological effects on pro-inflammatory cytokines. One key target is NF-κB, a transcription factor that regulates the expression of multiple pro-inflammatory cytokines, including Il-1β and Il6^50^. The mechanism of MON ultimately leads to a broader anti-inflammatory effect. MON has been approved for the management of asthma and alleviation of seasonal allergic symptoms^49,51^. We found that MON appeared to have a beneficial effect against cognitive deficits in 7.5- and 15-month-old AD mice. Previous studies have shown that MON can attenuate the cognitive deficits and AD hallmarks, including neuroinflammatory cytokines, Aβ levels, and oxidative stress makers^37,51^. Moreover, MON also plays a beneficial effect in hippocampal impairment induced by transient global cerebral ischemia, kainic acid, quinolinic acid/malonic acid and streptozotocin induced cognitive dysfunction and neurotoxicity^20,52–54^. Its pharmacological action is mainly involved in regulating the generation of inflammatory mediators^20,55^. Our findings are consistent with the main pathway through which MON exerts its efficacy. Sasapyrine is another anti-inflammatory drug that exerts its anti-inflammatory effects mainly by inhibiting cyclooxygenase (COX)-1 and COX-2, leading to a decrease in the levels of proinflammatory cytokines and associated symptoms^56^. Our results indicated a tendency of sasapyrine to rescue young AD flies, although the difference was not significant. This might reflect that only certain drug, such as the CysLT1R antagonist MON, is effective in the early stage of AD. The variation of drug efficacy could be due to their distinct acting mechanisms.

In the central nervous system (CNS), the proinflammatory cytokines can be neuroprotective or pathological^57,58^. The Aβ deposits can be both a cause and a consequence of proinflammatory cytokines in AD. A model proposed the feed-forward loop in which Aβ indirectly contributes to the activation of proinflammatory cytokines, and inflammation can increase Aβ accumulation and tau-mediated neurodegeneration^59^. In parallel with this model, proinflammatory cytokines are involved in the maintenance of activated microglia to reduce Aβ accumulation by increasing its phagocytosis^60^. Many neuroprotective effects of proinflammatory cytokines have been reported. For example, TNFα has been reported to protect against glutamate, free radical, and Aβ toxicity in enriched cultures of primary neurons^61^. Evidence has indicated that Il-1β may play a beneficial role in limiting AD pathology, and sustained overexpression of Il-1β reduces Aβ-related pathology by modulating microglia-dependent plaque degradation or promoting non-amyloidogenic APP cleavage in an AD mouse model and in cell culture^62–64^. Thus, proinflammatory cytokines might play complex roles in AD pathogenesis. Our findings indicated no significant difference in the expression level of AMPs in aged AD flies and proinflammatory cytokines in aged AD mice, compared to their aged controls. Our findings thus strongly suggest that the homeostatic status of inflammation in aged AD animals may involve a delicate equilibrium between neurotoxicity and neuroprotection mechanisms.

In the present study, we investigated the similarity of molecular features between different ages of AD mice and patients. The analysis focused solely on the number of genes among the differentially expressed genes in mice that exhibited concordant and discordant changes with those in patients. However, there were no comparative results for genes from other model organisms and AD patients, mainly due to the limited availability of multi-omics data for aged AD model organisms. Moreover, we cannot exclude the possibility that MON and sasapyrine administration would affect the expression level of anti-inflammatory cytokines. However, it would not alter our conclusion that the beneficial effect is limited to the proinflammatory efficacy induced by MON.

## Conclusions

In summary, the findings of the current study indicated that the aged AD animals better replicate the molecular characteristics of symptomatic patients. The distinct inflammatory status of AD animal models at different ages might lead to variations in anti-inflammatory drug efficacy. These findings underscore the importance of emphasizing disease progression in AD model research and drug development, while considering the pathological status of the models and patients to obtain more reliable research findings and drug efficacy data.

## Electronic supplementary material

Below is the link to the electronic supplementary material.

Supplementary Material 1

## Data Availability

All data generated in this research are available from the corresponding author on reasonable request.
