# Supplementary material for: The anti-inflammatory drug Montelukast ameliorates cognitive deficits by rescuing the inflammatory levels in young AD animal models
Source: Sci Rep. 2025 Apr 13;15:12720. doi: 10.1038/s41598-025-91785-4 (PMC11994820; doi:10.1038/s41598-025-91785-4)
Supplement: Supplementary file 1 — Supplementary Material 1 [file 41598_2025_91785_MOESM1_ESM.pdf]

The anti-inflammatory drug Montelukast ameliorates cognitive deficits by rescuing the inflammatory levels in young AD animal models

Mengnan Wu[1,†], Yan-Fen Chen[2, †], Wei Yao[2], Siyan Zhou[1], Zuolei Xie[2], Ye Tao[2], Yi Zhong[1,*], Weiwei Ma[1,2,*]

[1] School of Life Sciences, Tsinghua University, Beijing 100084, China
[2] Beijing Joekai Biotechnology LLC, Beijing 100094, China
[3] These authors contributed equally.

* Corresponding author.
Email: maweiwei@joekai.com (W. Ma)
zhongyi@mail.tsinghua.edu.cn (Y. Zhong)

The PDF file includes:

Supplementary Figure 1 to 4
Supplementary Table 1

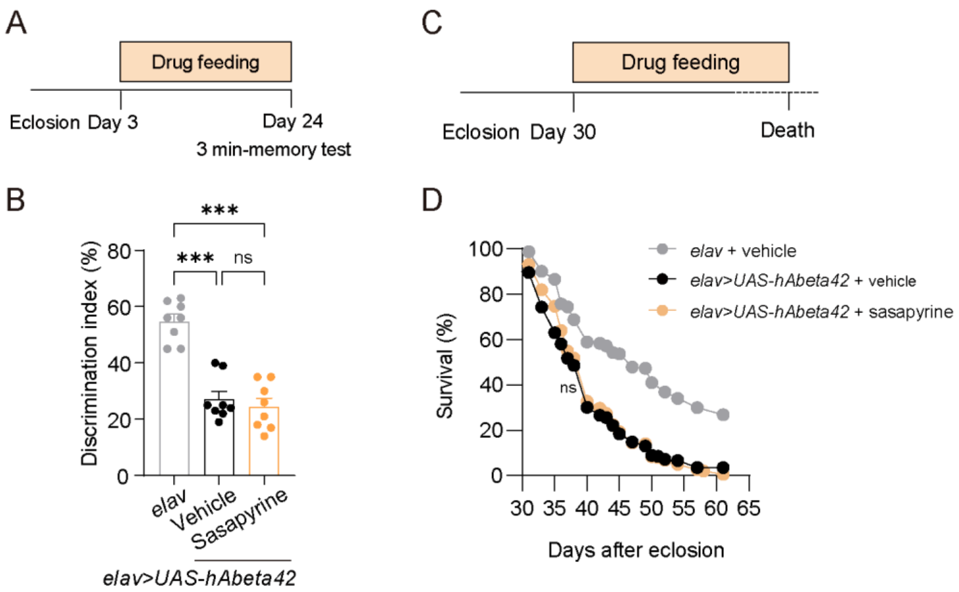

Figure S1. The treatment effect of sasapyrine on performance index and longevity. (A-B) Schemic (**A**) and quantification (**B**) of the performance index for flies treated with sasapyrine. n = 8 vials of flies per group, 100 flies per vial (One-way ANOVA, $F_{2,21} = 67.96$, $P < 0.001$). **(C-D)** Schematic (**C**) and survival probability (**D**) of flies treated with sasapyrine. *elav* + vehicle, n = 173; *elav>UAS-hAbeta 42* + vehicle, n = 222; *elav>UAS-hAbeta 42* + sasapyrine, n = 262 flies (log-rank Mantel-Cox test, $\chi^2 = 91.81$, $P < 0.001$). Data are expressed as mean + SEM. *** $P < 0.001$; ns, not significant.

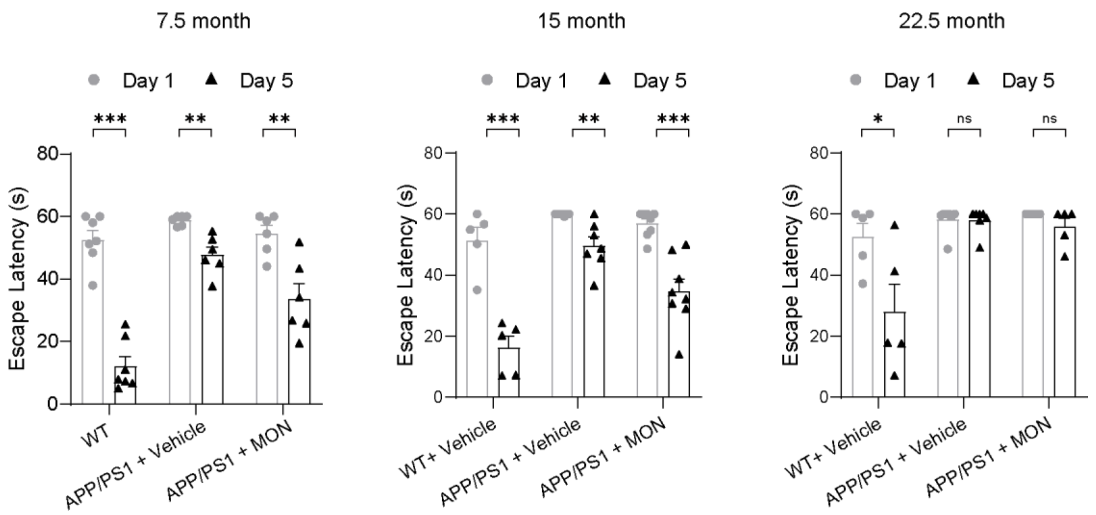

Figure S2. Comparation of escape latency on day 0 and 5. (**A**) Escape latency for 7.5-month-old mice. WT + Vehicle: n = 7, t_{12} = 15.58, $P < 0.0001$; APP/PS1 + Vehicle: n = 6, t_{10} = 4.79, $P = 0.0007$; APP/PS1+ MON: n = 6, t_{10} = 5.33, $P = 0.0003$. (**B**) Escape latency of 15-month-old mice. WT + Vehicle: n = 6, t_{10} = 13.55, $P < 0.0001$; APP/PS1 + Vehicle: n = 7, t_{12} = 3.60, $P = 0.004$; APP/PS1+ MON: n = 8, t_{14} = 6.26, $P < 0.0001$. (**C**) Escape latency for 22.5-month-old mice. WT + Vehicle: n = 5, t_8 = 3.54, $P = 0.008$; APP/PS1 + Vehicle: n = 7, t_{12} = 1.34, $P = 0.20$; APP/PS1 + MON: n = 5, t_{11} = 1.59, $P = 0.14$. Unpaired student's *t*-test. Data are expressed as mean + SEM. $^{**}P < 0.01$; $^{***}P < 0.001$; ns, not significant.

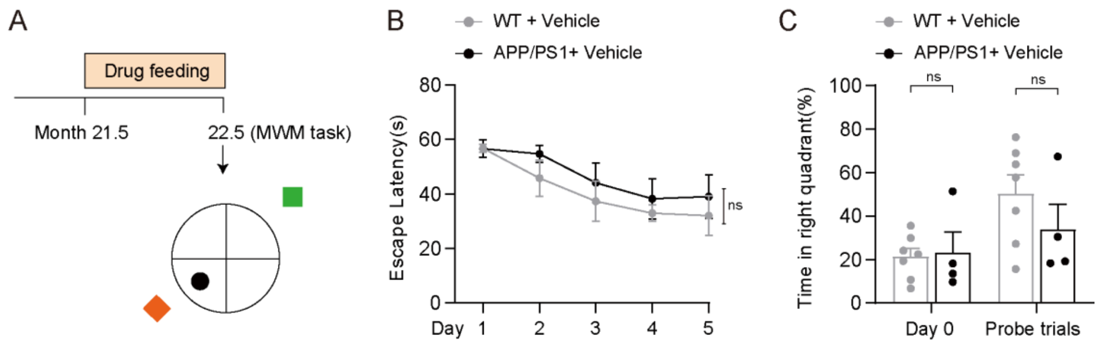

Figure S3. The learning and memory ability at 22.5-month-old mice could not be assessed using MWM paradigm with two cues. (**A**) Schematic illustration of the MWM task of 22.5-month-old AD mice. Two cues were placed on opposite quadrants as the cue for spatial reference. (**B**) The escape latency during training (Repeated measures of ANOVA test with Tukey HSD post-hoc multiple comparisons: day, $F_{4,36}$ = 7.97, P = 0.0001; group, $F_{1,9}$ = 0.68, P = 0.43; day x group, $F_{4,36}$ = 0.27, P = 0.90). (**C**) The amount of time spent in the right quadrant when the platform was absent of 22.5-month-old AD mice. (Unpaired Student's *t*-test: Day 0, t_9 = 1.00, P = 0.34; Probe trail, t_9 = 0.71, P = 0.49). Data are expressed as mean + SEM. ns, not significant.

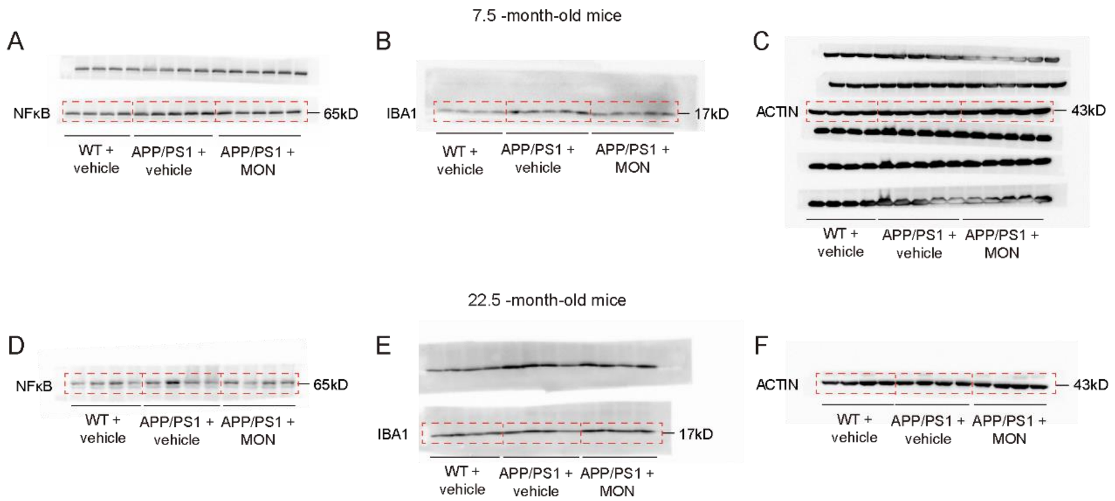

Figure S4. Uncropped immunoblots from figure 3B and F. (**A-C**) The uncropped immunoblots of 7.5-month-old mice. (**A**) The Immunoblots of NFκB (inset). The top image was used for other experiments. (**B**) The immunoblots of IBA1 (inset). (**C**) The immunoblots of ACTIN (inset). The other images were used for other experiments. (**D-F**) The uncropped immunoblots of 22.5-month-old mice. (**D**) The immunoblots of NFκB (inset). (**E**) The immunoblots of IBA1 (inset). The top image was used for other experiments. (**F**) The immunoblots of ACTIN (inset). Images were taken by miniChemi 610 PLUS (Beijing Sage Creation).

Table S1. Sequence of primers used for qRT-PCR analysis

**Gene (drosophila)**	**Sequence (5' to 3')**	
	**Forward**	**Reverse**
Dpt	GCTGCGCAATCGCTTCTACT	TGGTGGAGTGGGCTTCATG
Drs	CGTGAGAACCTTTTCCAATATGATG	TCCCAGGACCACCAGCAT
Mtk	CGTCACCAGGGACCCATTT	CCGGTCTTGGTTGGTTAGGA
Rp49	GACGCTTCAAGGGACAGTATCTG	AAACGCGGTTCTGCATGAG

**Gene (mouse)**	**Sequence (5' to 3')**	
	**Forward**	**Reverse**
Il-1β	CAACCAACAAGTGATATTCTCCATG	GATCCACACTCTCCAGCTGCA
Il-6	TAGTCCTTCCTACCCCAATTTCC	TTGGTCCTTAGCCACTCCTTC
18s rRNA	TAAGTCCCTGCCCTTTGTACACA	GATCCGAGGGCCTCACTAAAC
